# Assessment of Awareness, Knowledge, and Self-Reported Prevalence of Toxoplasmosis in the Tropical Zone of Saudi Arabia: A Cross-Sectional Study

**DOI:** 10.3390/tropicalmed10110323

**Published:** 2025-11-17

**Authors:** Hassan N. Moafa, Ahmad Mobarki, Sultan Moafa, Ziyad Asiri, Ahmed Hadadi, Osama M. Abualgasem, Rama M. Chandika, Jobran M Moshi, Ashwaq M Al Nazawi, Raad Shibli, Hammad Ali Fadlalmola

**Affiliations:** 1 Department of Public Health, College of Nursing and Health Sciences, Jazan University, Jazan 82912, Saudi Arabia; amobarki@stu.jazanu.edu.sa (A.M.); smoafa@stu.jazanu.edu.sa (S.M.); zasiri@stu.jazanu.edu.sa (Z.A.); ahadadi@stu.jazanu.edu.sa (A.H.); oabualgasem@stu.jazanu.edu.sa (O.M.A.); anazawi@jazanu.edu.sa (A.M.A.N.); 2 Clinical Nutrition Department, College of Nursing and Health Sciences, Jazan University, Jazan 45142, Saudi Arabia; rchandika@jazanu.edu.sa; 3 Department of Medical Laboratory Technology, College of Nursing and Health Sciences, Jazan University, Jazan 82912, Saudi Arabia; jmoshi@jazanu.edu.sa; 4 Laboratory Department, Jazan University Hospital, Jazan University, Jazan 82911, Saudi Arabia; gwlnw@hotmail.com; 5 Department of Community Health Nursing, Nursing College, Taibah University, Madinah 42353, Saudi Arabia; hafadlelmola@taibahu.edu.sa

**Keywords:** toxoplasmosis, *Toxoplasma gondii*, awareness, knowledge, zoonotic diseases, risk factors, Jazan region, Saudi Arabia

## Abstract

Background: Toxoplasmosis, caused by the parasite *Toxoplasma gondii*, is a zoonotic disease that poses significant health risks to immunocompromised individuals, pregnant women, and infants. Transmission occurs primarily through infected cat feces or contaminated food. Awareness of transmission routes, prevention strategies, and health consequences remains limited in high-prevalence humid regions such as Jazan, Saudi Arabia. Methods: This cross-sectional study was conducted in Jazan, Saudi Arabia, between April and May 2025, surveying 485 adults using a five-section questionnaire covering demographics, knowledge, practices, medical history, and recommendations. The survey was distributed in both English and Arabic. Self-reported previous diagnoses were used to estimate prevalence, with risk factors presented as frequencies and percentages. Binary logistic regression analyzed categorical variables, and independent *t*-tests assessed continuous variables to identify predictors of awareness and knowledge regarding toxoplasmosis. Results: Participants comprised 58.6% females, 97.3% Saudis, and 69.1% individuals aged ≤30 years; 49.7% had heard of toxoplasmosis. Females (adjusted odds ratio [AOR]: 1.67, 95% CI: 1.13–2.5, *p* < 0.01) and those >30 years old (AOR: 1.8, 95% CI: 0.80–4.29, *p* > 0.05) demonstrated greater awareness and knowledge, though this was not statistically significant. No significant differences were observed based on marital status (*p* > 0.05). Risk behaviors included consuming unwashed fruits and vegetables (27.6%) and unpasteurized dairy products (28.2%), with 62.7% always washing hands after handling raw meat or soil. Cat ownership (20.6%) was not associated with knowledge (*p* = 0.97). Self-reported diagnosis prevalence was 1.9%. Conclusions: Low awareness and prevalent risky behaviors underscore the urgent need for targeted public health education interventions focusing on hygiene practices and zoonotic disease prevention in Jazan. Serological studies are recommended to obtain more accurate prevalence estimates and guide evidence-based interventions.

## 1. Introduction

*Toxoplasma gondii*, an obligate intracellular protozoan parasite of the phylum Apicomplexa, is the causative agent of toxoplasmosis, a zoonotic infection with substantial global public health implications [1]. Members of the family Felidae, including domestic cats, serve as the definitive hosts, with transmission to humans occurring primarily through ingestion of tissue cysts in undercooked meat, exposure to oocysts shed in cat feces, or vertical transmission from mother to fetus [2,3]. Although infection is often asymptomatic in immunocompetent hosts, severe ocular or neurologic complications may occur in a subset of cases, and rarely, life-threatening manifestations such as pneumonia, myocarditis, or multi-organ failure have been reported, potentially due to virulent strains. It can cause severe complications in vulnerable populations, including ocular toxoplasmosis (retinochoroiditis), encephalitis in immunocompromised individuals, and congenital disabilities such as hydrocephalus or intellectual disability in neonates [4,5].

Building on this, global seroprevalence varies considerably, ranging from 10% in some high-income countries to over 80% in certain tropical regions. This variation is influenced by climatic conditions—particularly high humidity and temperature that favor oocyst survival—as well as sociocultural factors including consumption of raw or undercooked meat and close contact with cats [6,7].

In Saudi Arabia, toxoplasmosis seroprevalence exhibits marked regional and demographic variation. A recent systematic review and meta-analysis synthesizing data from 30 studies conducted between 1994 and 2023 estimated a national pooled IgG seroprevalence of 27.5% and IgM seroprevalence of 2.2% among 20,699 participants [8]. Regional IgG prevalence was highest in the Eastern (37.2%) and Western (32.8%) regions, with lower rates in the Northern (15.4%) and Southern (28.7%) regions. Age-specific analysis revealed peak prevalence in the 31–45 years age group (32.5%). While the water source showed a minor variation in reported prevalence (33.5% vs. 29.4%), this difference was not statistically significant and only illustrates regional variability. Among pregnant women—a particularly high-risk group—IgG seroprevalence across 20 studies involving 15,797 individuals was 28.7%, with IgM at 2.7% [8].

Specific studies from various regions illustrate this heterogeneity. In the Eastern region, Yanez et al. (1994) reported 37.5% IgG seroprevalence in 784 general population participants from Al-Ahsa [9], while Al-Mulhim et al. (2001) found 39.4% IgG and 0.75% IgM in 175 pregnant women from Al-Khobar using microparticle enzyme immunoassay [10]. Western region studies include Ghazi et al. (2002), who documented 35.6% IgG in 926 pregnant women from Mecca using ELISA [11], and Al-Harthi et al. (2006), reporting 29.9% IgG and 5.58% IgM in 197 pregnant women [12].

In the Southern region, particularly Jazan, studies have reported variable results. Eisa et al. (2013) found 40.8% IgG and 4% IgM in 174 general participants [13]; Aqeely et al. (2014) reported 20% IgG and 6.2% IgM in 195 pregnant women [14]; and Eida et al. (2015) documented 14.6% IgG in 226 pregnant women using ELISA and PCR [15]. Additional contributions from other regions include Almogren et al. (2011), who reported 37.9% IgG in 2176 pregnant women from Riyadh [16], and Al-Hakami et al. (2020), finding 27.4% IgG in 190 pregnant women from Abha [17].

Despite this serological evidence indicating moderate national prevalence—comparable to the global average of 30–40% [6]—significant knowledge gaps persist regarding awareness and preventive behaviors, particularly in understudied regions such as Jazan. Recent global systematic reviews have highlighted rising seroprevalence in humid tropical areas attributed to climate change, further emphasizing Jazan’s vulnerability [4]. Jazan’s humid tropical climate and predominantly agricultural lifestyle may facilitate environmental transmission. Nevertheless, only three studies have specifically examined the Jazan region, revealing inconsistent prevalence rates (14.6–40.8%) and limited focus on community knowledge and risk behaviors [13,14,15].

Furthermore, recent climatological analyses have revealed significant seasonal shifts in Jazan’s rainfall patterns, with precipitation peaks transitioning from autumn to summer and increasing in intensity over time [18]. The Saudi Center for Meteorology forecasts that Jazan will experience a year-round humid tropical climate [19], potentially exacerbating risks of zoonotic diseases including toxoplasmosis. The paucity of data on public awareness and understanding hampers effective public health responses, as low awareness frequently leads to risky practices such as consuming unwashed produce or inadequate hygiene around cats. Addressing this gap is critical, given that enhanced awareness could substantially reduce infection incidence in high-risk groups such as pregnant women, among whom congenital toxoplasmosis remains a pressing concern [5].

This study aimed to investigate awareness, knowledge, and preventive practice adoption regarding toxoplasmosis among adults in the Jazan region of Saudi Arabia, while estimating self-reported prevalence of prior diagnoses in high-risk individuals. We sought to identify factors associated with toxoplasmosis awareness and knowledge, thereby providing evidence to inform targeted public health interventions in this vulnerable region.

## 2. Materials and Methods

### 2.1. Study Settings

The study was conducted in the Jazan region, located in southwestern Saudi Arabia at the border with Yemen. Jazan is a humid southern region approaching tropical conditions according to recent climate analyses, but formally arid under the Köppen classification. Jazan has a population of approximately 1.5 million distributed across 16 provinces, each characterized by distinct geographical features. The majority of the population resides in rural areas, with a balanced sex ratio of approximately 1:1 [20].

### 2.2. Study Design

This cross-sectional study investigated awareness, knowledge, and preventive practices related to toxoplasmosis among the adult population in the Jazan region of Saudi Arabia. Self-reported data were utilized to estimate the prevalence of prior diagnoses among high-risk individuals identified through the questionnaire. The study adheres to the Strengthening the Reporting of Observational Studies in Epidemiology (STROBE) guidelines for cross-sectional studies (Supplementary Table S1).

### 2.3. Study Period

Data collection was conducted between April 2025 and May 2025.

### 2.4. Sample Size Calculation

Sample size was calculated using the following formula for estimating prevalence:

*n* = (Z^2^ × P × (1 − P))/e^2^

where

- Z = 1.96 (corresponding to 95% confidence level);
- P = 0.2952 (estimated prevalence based on national seroprevalence data [8]);
- e = 0.05 (margin of error).

This calculation yielded a minimum required sample size of 320 participants. To account for potential non-responses, incomplete questionnaires, and to ensure adequate statistical power, a total of 485 participants were recruited and included in the final analysis.

### 2.5. Ethical Approval

Ethical approval was obtained from the Scientific Research Ethics Committee at Jazan University (protocol code HAPO-10-Z-001, registration record number REC-46/09/1395, approved 4 March 2025). The study was conducted in accordance with the Declaration of Helsinki. The principles of privacy and confidentiality were strictly maintained throughout the research. All participants provided informed consent and were informed of their rights, including the freedom to decline participation or withdraw at any time without consequence.

### 2.6. Data Collection Instrument

A structured questionnaire was developed based on comprehensive literature reviews and expert consultation to ensure content validity and relevance to the local context. A pilot study involving 25 individuals from the Jazan region was conducted to assess the instrument’s reliability, yielding a Cronbach’s alpha coefficient of 0.78 for the combined responses from English and Arabic questionnaires, indicating acceptable internal consistency [21]. Further, on the whole sample, Cronbach’s alpha coefficient for the awareness and knowledge items was obtained as 0.81, indicating consistent responses among participants.

The final questionnaire comprised 47 items divided into five sections: (1) Demographic information (nationality, age, gender, marital status, residence, education level, occupation, monthly income). Marital status was categorized as married or unmarried, with unmarried including single, divorced, and widowed participants. (2) Knowledge about toxoplasmosis transmission and prevention (4 true/false questions). (3) Attitudes and practices related to toxoplasmosis, including dietary habits and hygiene practices assessed using a 5-point Likert scale (1 = Never, 2 = Rarely, 3 = Sometimes, 4 = Often, 5 = Always). (4) Medical history and preventive measures. (5) Recommendations regarding preferred health information sources and awareness-raising suggestions.

Participants identified as high-risk (pregnant or planning pregnancy, history of miscarriage, or immunocompromised based on self-reported symptoms) were asked about previous toxoplasmosis diagnoses and testing history. Serological testing could not be performed due to institutional budget constraints. Questionnaire completion time ranged from 8 to 12 min. The complete questionnaire is provided as Supplementary File S1.

The questionnaire was distributed via social media platforms (WhatsApp, Twitter, and Facebook) and as printed copies in community centers and healthcare facilities to ensure diverse demographic representation. Both English and Arabic versions were available to accommodate participants’ language preferences. Data were collected continuously from April to May 2025 via printed questionnaires (40% of responses) and online platforms (60%). No compensation was provided to the participants.

### 2.7. Data Management and Statistical Analysis

Data were analyzed using R version 4.3.2 (R Core Team, Vienna, Austria) within the RStudio environment (version 2024.12.1; Posit Software, PBC, Boston, MA, USA). Descriptive statistics including mean ± standard deviation for continuous variables and frequencies with percentages for categorical variables (demographics, risk behaviors) were computed. Age was dichotomized at ≤30 versus >30 based on the sample median and national demographics. Income was categorized at less than 5000 SAR (low) versus greater than or equal to 5000 SAR (Saudi Arabian Riyal) in accordance with established socioeconomic standards and thresholds.

Awareness was defined as having heard of toxoplasmosis (binary: yes/no). Knowledge was quantified as a continuous score (0–4) based on correct responses to four questions regarding transmission routes and prevention methods. Binary logistic regression was performed to assess associations between demographic factors and awareness, with odds ratios (ORs) and adjusted odds ratios (AORs) with 95% confidence intervals (CI) calculated for both univariate and multivariate models. Using stepwise selection, the demographic variables were included in the multivariable logistic regression model. Variables were retained in the final model if they met a significance threshold of *p* < 0.05. Variance inflation factors are considered to be below 5 for low collinearity. Internal validation was performed using bootstrapping with 1000 resamples to assess potential overfitting. Independent *t*-tests were employed to compare mean knowledge scores across demographic categories. Assumptions for *t*-tests (normality and homogeneity of variance) were verified using Shapiro–Wilk and Levene’s tests, respectively. Relying on the central limit theorem, our large sample justifies the normality assumptions. Additionally, the t-test provides greater statistical power than non-parametric tests.

Statistical significance was set at *p* < 0.05 for all analyses. For logistic regression, multivariate models were constructed including all demographic variables to calculate adjusted odds ratios, controlling for potential confounding effects.

## 3. Results

### 3.1. Demographic Characteristics of Participants

A total of 485 individuals participated in the study. Females comprised 58.6% (n = 284) of the sample, with 97.3% (n = 472) being Saudi nationals. Over two-thirds (69.1%, n = 335) of participants were aged ≤30 years, and 66.4% (n = 322) were unmarried. More than half (58.1%, n = 282) resided in cities, and 77.7% (n = 377) had attained a bachelor’s degree or higher educational qualification. A substantial proportion (70.5%, n = 342) were unemployed (including students and homemakers), and 59.4% (n = 288) reported a monthly income of <5000 SAR. Detailed demographic characteristics are presented in Table 1.

### 3.2. Factors Associated with Toxoplasmosis Awareness

Overall, 241 (49.7%) participants reported having heard of toxoplasmosis. Univariate analysis revealed that females demonstrated significantly higher awareness (53.5%) compared to males (44.3%; crude odds ratio [COR]: 1.45, 95% CI: 1.01–2.09, *p* = 0.04). This association remained significant in the multivariate model (AOR: 1.67, 95% CI: 1.13–2.50, *p* = 0.01). Participants aged >30 years showed marginally higher awareness (56.0%) than those ≤30 years (46.9%; COR: 1.44, 95% CI: 0.98–2.13, *p* = 0.06), although this difference did not reach statistical significance. This association also did not reach statistical significance in the adjusted model (AOR: 1.8, 95% CI: 0.80–4.29, *p* = 0.16).

Non-Saudi participants showed slightly higher awareness (61.5%) compared to Saudi nationals (49.4%), though this difference was not statistically significant (AOR: 2.20, 95% CI: 0.68–7.78, *p* = 0.19). No statistically significant associations were observed for marital status, residence, education level, occupation, or monthly income in either crude or adjusted models (all *p* > 0.05). Detailed results of the logistic regression analysis are presented in Table 2.

### 3.3. Knowledge Scores Across Demographic Groups

Knowledge was quantified using a score ranging from 0 to 4, based on correct responses to four questions about transmission routes and prevention methods. The mean knowledge score for the entire sample was 1.9 ± 1.5 (range: 0–4).

Females demonstrated significantly higher mean knowledge scores (2.2 ± 1.5) compared to males (1.6 ± 1.5; t = 4.50, *p* < 0.001, mean difference [MD] = 0.6, 95% CI: 0.4–1.0). Similarly, participants aged >30 years had significantly higher knowledge scores (2.1 ± 1.6) than those ≤30 years (1.8 ± 1.5; t = 2.08, *p* = 0.04, MD = 0.3, 95% CI: 0.0–0.8).

No significant differences in knowledge scores were observed for nationality (*p* = 0.78), marital status (*p* = 0.06), residence (*p* = 0.27), education level (*p* = 0.57), occupation (*p* = 0.24), or monthly income (*p* = 0.54). Detailed results of the knowledge score comparisons are presented in Table 3.

### 3.4. Sources of Information on Toxoplasmosis

**Figure 1:**
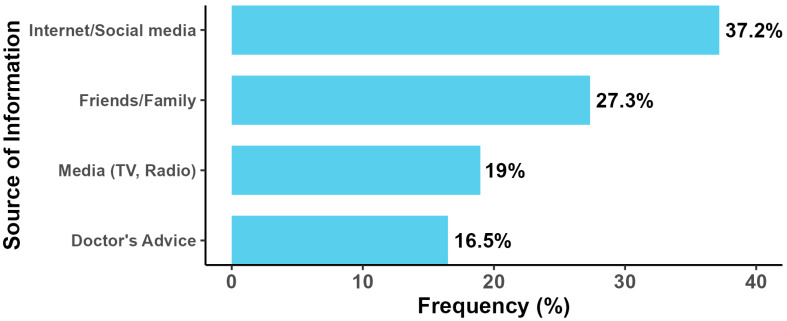
Source of Information for Participants.

Among the 241 participants who reported awareness of toxoplasmosis, the internet and social media were the most frequently cited sources of information (37.2%, n = 90), followed by healthcare professionals/doctors’ advice (24.9%, n = 60), friends or family (18.7%, n = 45), traditional media including television and radio (12.4%, n = 30), and educational institutions (6.6%, n = 16). These findings are illustrated in Figure 1.

### 3.5. Self-Reported Risk Behaviors

Risk behaviors related to potential toxoplasmosis exposure were assessed using a 5-point Likert scale (Table 4). Consumption of undercooked meat (steaks, lamb chops) was reported by 11.8% of participants at frequencies ranging from rarely to always (mean score: 1.2 ± 0.7). Raw or undercooked fish consumption was reported by 9.9% (mean score: 1.2 ± 0.5). A substantial proportion reported consuming unwashed fruits and vegetables (27.6%, mean score: 1.5 ± 0.9) and unpasteurized dairy products such as raw milk and soft cheese (28.2%, mean score: 1.5 ± 0.9).

Regarding hygiene practices, 62.7% of participants reported “always” washing their hands after handling raw meat or soil (mean score: 3.9 ± 1.6), while 53.2% reported “always” washing fruits and vegetables before consumption (mean score: 3.8 ± 1.5). These findings indicate substantial room for improvement in preventive hygiene practices.

### 3.6. Cat Ownership and Related Practices

Cat ownership was reported by 100 participants (20.6%). Among cat owners, 88.0% (n = 88) reported regular litter box cleaning, 63.0% (n = 63) used gloves during cleaning, and 87.0% (n = 87) washed their hands after cleaning. However, only 46.0% (n = 46) reported that their cats had been vaccinated against toxoplasmosis, while 37.0% (n = 37) reported no vaccination, and 17.0% (n = 17) were unsure of vaccination status.

Cat ownership showed no significant association with knowledge scores (mean score for owners: 1.9 ± 1.6 vs. non-owners: 1.9 ± 1.5; t = 0.04, *p* = 0.97, 95% CI for MD: −0.5 to 0.4), suggesting that pet ownership alone does not translate to increased knowledge about zoonotic disease transmission without targeted education.

### 3.7. Self-Reported Symptoms and Medical History

Among all participants, self-reported symptoms potentially associated with toxoplasmosis included fever, fatigue, muscle aches, or headache (45.0%, n = 218), swollen lymph nodes (30.0%, n = 146), sore throat, cough, or shortness of breath (35.0%, n = 170), rash (25.0%, n = 121), eye irritation including redness or photosensitivity (20.0%, n = 97), and neurological symptoms such as confusion, seizures, or difficulty concentrating (10.0%, n = 49). However, these symptoms are non-specific and cannot be definitively attributed to toxoplasmosis without laboratory confirmation.

Among female participants (n = 284), 20.0% (n = 57) reported being currently pregnant or planning pregnancy, and 15.0% (n = 43) reported a history of miscarriage or stillbirth. Among those with miscarriage history, the mean number of events was 1.5 (range: 1–4), with the majority occurring during the first trimester. Birth defects in offspring were reported by 5.0% (n = 14) of female participants.

Self-reported prior diagnosis of toxoplasmosis was documented in 1.9% (n = 9) of participants, substantially lower than serological prevalence reported in previous studies from the region [13,14,15]. Only 2.3% (n = 11) reported ever being tested for toxoplasmosis, with most tests conducted within the preceding 1–2 years. Among those diagnosed, 5 participants (1.0% of total sample) reported receiving medication, most commonly pyrimethamine combined with sulfadiazine.

### 3.8. Recommendations for Awareness Enhancement

When asked about the need for increased public health awareness regarding toxoplasmosis, 80.0% (n = 388) of participants responded affirmatively. Preferred sources for accurate health information included clinics and hospitals (40.0%, n = 194), internet and medical websites (30.0%, n = 146), social media (20.0%, n = 97), and traditional media including television and radio (10.0%, n = 49).

Interest in undergoing blood testing for toxoplasmosis was expressed by 60.0% (n = 291) of participants, indicating substantial willingness to engage with screening programs if made available.

## 4. Discussion

The present study reveals a moderate awareness rate of 49.7% regarding toxoplasmosis in the Jazan region, with significant knowledge gaps when compared to serological evidence. The self-reported prior diagnosis of 1.9% starkly contrasts with the pooled national IgG seroprevalence of 27.5% [8] and regional studies reporting 14.6–40.8% prevalence in Jazan [13,14,15], suggesting either widespread undiagnosed cases or high prevalence of asymptomatic infections. Although our findings cannot be directly compared to serological data due to methodological differences, these studies [13,14,15] serve as important references for understanding the potential extent of toxoplasmosis in the region and highlight the need for integrated awareness and testing initiatives. This discrepancy aligns with the characteristically asymptomatic nature of acute toxoplasmosis in immunocompetent adults [3], where individuals may harbor the parasite unknowingly, thereby perpetuating transmission through unaddressed risk behaviors. Similar patterns have been documented in seroprevalence studies from other regions globally [22]. For example, a recent Brazilian study showed that seroprevalence among pregnant women in humid areas was 55.1% [23], while in Sudan—located on the western side of the Jazan region of Saudi Arabia—another recent meta-analysis estimated pooled seroprevalence at varying levels, influenced by environmental factors [24], underscoring the need for targeted awareness campaigns in similar humid areas, such as the Jazan region.

Our sociodemographic analyses provide important insights into factors influencing awareness and knowledge. Females demonstrated significantly elevated awareness (AOR: 1.67, 95% CI: 1.13–2.50, *p* = 0.01) and higher mean knowledge scores (2.2 ± 1.5 vs. 1.6 ± 1.5, *p* < 0.001) compared to males. This finding likely reflects greater exposure to health education during prenatal care and is consistent with studies showing heightened concern about toxoplasmosis among women of reproductive age [8,24]. Age was also significantly associated with knowledge (*p* = 0.04), mirroring national trends where prevalence peaks in the 31–45 years age group (32.5%) [8], potentially reflecting cumulative environmental exposure over time [6].

Interestingly, marital status showed no significant association with awareness or knowledge (*p* = 0.46), diverging from the hypothesis that married individuals, often with family responsibilities, may demonstrate greater health consciousness. This lack of association with marital status can be explained by Saudi culture, where people are most likely to share household exposure or have equal educational access within the Saudi community, where cultural norms emphasize relatives and family-based health practices regardless of marital status. In Saudi Arabia, family members collectively plan their lifestyles and even decide on access to secondary healthcare facilities. This suggests that, in the Jazan context, factors such as education level and media access may override marital influences on health knowledge, indicating that toxoplasmosis is more impacted by community-level factors than by individual marital dynamics [25].

The identified risk behaviors are concerning and align with known transmission routes. Consumption of unwashed fruits and vegetables (27.6%) and unpasteurized dairy products (28.2%) are established risk factors, particularly relevant to Jazan’s agricultural setting where soil contamination with oocysts is likely [8]. The finding that only 62.7% consistently wash hands after handling raw meat or soil highlights critical hygiene gaps in a humid tropical climate that favors oocyst survival and transmission [4]. These behavioral patterns underscore urgent needs for targeted food safety and hygiene education.

Cat ownership (20.6%) without significant knowledge correlation (*p* = 0.97) is particularly noteworthy, as cats are definitive hosts capable of shedding millions of oocysts [2]. The relatively low vaccination rate (46%) and suboptimal glove use (63%) during litter box cleaning indicate substantial educational gaps despite proximity to a primary transmission source. This aligns with findings from Eisa et al. (2013), who reported 40.8% prevalence in Jazan populations with animal contact [13]

Compared to previous awareness studies, our finding of 49.7% awareness represents a substantial improvement over earlier reports from Riyadh, where 79.2% of women were unaware of toxoplasmosis [26]. Comparative studies from other Saudi capital cities, such as Jeddah, where only 46.7% of pregnant women had heard of toxoplasmosis [27], and Riyadh, where only 20.8% of women were aware of the disease [26], highlight the need for nationwide education. Our study, along with those from Riyadh [26] and Jeddah [27], reinforces this necessity. Globally, seroprevalence among pregnant women remains high in humid areas [4], underscoring the urgency of targeted interventions in regions with high humidity, such as Jazan. This may reflect increasing national health education efforts and growing awareness of zoonotic diseases, particularly relevant given the emerging trend of pet ownership among Saudi women. However, awareness without accurate knowledge of transmission routes and prevention methods remains inadequate to effectively reduce transmission risk.

The symptom reporting in our study (e.g., 45% fever/fatigue, 30% swollen lymph nodes) overlaps with non-specific manifestations of acute toxoplasmosis [1], though self-reported data preclude definitive causal attribution. Among female participants, the 15% with a history of miscarriage or stillbirth is particularly concerning, given that only 9% of this subgroup reported awareness of toxoplasmosis as a possible contributing factor based on cross-tabulation analysis. This discrepancy, along with national data showing 28.5% IgG seroprevalence in pregnancies with abnormal outcomes [8], indicates potential gaps in antenatal screening and education, which could lead to preventable adverse events. Addressing this through integrated toxoplasmosis awareness in postnatal and antenatal care could improve prevention strategies and birth outcomes in Saudi Arabia, particularly in humid high-risk regions like Jazan.

Recent studies from other Saudi cities provide context for our findings. Alhussainy et al. documented 5.6% toxoplasmosis seropositivity among non-pregnant women in a metropolitan area [28], which aligns with our self-reported prevalence of 1.9%, though both likely underestimate the true burden given the predominantly asymptomatic nature of infection. Both studies underscore the substantial gap between serological evidence and clinical diagnosis/awareness.

Our findings complement existing serological studies from Jazan [13,14,15], which reported variable prevalence (14.6–40.8%), possibly due to methodological differences (ELISA vs. PCR), different study populations, and temporal variations. The present study uniquely contributes by documenting awareness, knowledge, and risk behaviors, thereby identifying specific targets for public health intervention rather than solely estimating prevalence.

The high interest in testing (60%) and strong support for increased awareness campaigns (80%) indicate substantial community engagement potential. Participants’ preferred information sources—clinics and hospitals (40%), internet (30%), and social media (20%)—should guide intervention design to maximize reach and effectiveness. The predominant reliance on internet and social media (37.2%) as current information sources suggests opportunities for evidence-based digital health education campaigns.

### Strengths and Limitations

This study’s strengths include a representative sample size exceeding the calculated minimum requirement, comprehensive assessment of multiple domains (awareness, knowledge, practices, and medical history), and alignment with national meta-analysis findings [8]. The use of both Arabic and English questionnaires enhanced accessibility and demographic representativeness.

However, several limitations merit consideration. First, reliance on self-reported data introduces potential recall and social desirability bias, particularly regarding hygiene practices and symptom reporting. Second, the convenience sampling strategy, while pragmatic, may have introduced selection bias favoring more educated respondents with internet access, potentially overestimating awareness in the general population. In our convenience study, we could not assess non-response bias because the total number of exposed individuals or invited participants was not recorded. Selection bias may also result from a skewed sample, such as 78% highly educated and 70% unemployed individuals, which could overrepresent certain groups. This was mitigated by diverse recruitment methods (print and online), but larger-scale studies recruiting participants from multiple locations nationwide are necessary to minimize bias further. Third, the absence of serological validation prevents definitive prevalence estimation and verification of self-reported diagnoses. The 1.9% self-reported prevalence substantially underestimates the likely true burden based on previous serological studies [13,14,15]. Moreover, our reporting should not be considered a direct measure of the study period prevalence of toxoplasmosis in Jazan, as serological testing is essential for accurate reporting. The lack of serological data hampers the ability to distinguish between chronic (IgG) and acute (IgM) infections, thereby limiting conclusions about the timing of Toxoplasma parasite acquisition relative to recent climate changes in Jazan. Furthermore, this study does not include new climatological measurements or analyses; references to Jazan’s climate (e.g., humid or emerging tropical features) are contextual, derived from cited external sources [18,19], and aim to underscore potential humidity-related risks for toxoplasmosis transmission, without implying a formal reclassification from its arid Köppen–Geiger BWh status [29]. Fourth, the cross-sectional design precludes establishing temporal relationships or causal inferences between risk factors and outcomes. Finally, the study was conducted during a brief two-month period, which may not capture seasonal variations in exposure risk or awareness.

Future research should incorporate serological testing to accurately determine seroprevalence, conduct longitudinal follow-up to assess incident infections and risk factor contributions, and implement randomized controlled trials to evaluate the effectiveness of targeted educational interventions. Furthermore, such studies are warranted to explore potential associations between evolving climatic conditions and infection incidence in the region. Geographic expansion to other understudied regions of Saudi Arabia would enhance generalizability and enable regional comparisons, while employing probability sampling could better quantify response rates and minimize biases.

## 5. Conclusions

Half of the population in Jazan was aware of toxoplasmosis, but significant knowledge gaps and risky behaviors remain prevalent, such as poor food hygiene, consuming unpasteurized dairy, and unsafe pet care practices. The low self-reported prevalence (1.9%) does not reflect the true burden and contrasts with higher serological estimates from previous studies, underscoring the need for better diagnostic methods. Older adults and women showed greater knowledge. Public health policymakers should focus on targeted education about hygiene and zoonotic diseases, incorporate toxoplasmosis screening into antenatal care in primary health services for high-risk groups, and utilize clinics, online platforms, and social media to improve access to serological testing. Future research should include serological studies to accurately determine prevalence, longitudinal assessments of infection risks, and evaluations of intervention effectiveness to support evidence-based strategies in hot, humid regions like Jazan.

## Figures and Tables

**Table 1:** Demographic characteristics of study participants (n = 485).

Demographic Factor	Category	n (%)
Gender	Male	201 (41.4)
	Female	284 (58.6)
Nationality	Saudi	472 (97.3)
	Non-Saudi	13 (2.7)
Age	≤30 years	335 (69.1)
	>30 years	150 (30.9)
Marital Status	Unmarried	322 (66.4)
	Married	163 (33.6)
Residence	City	282 (58.1)
	Village	203 (41.9)
Education Level	High School or less	108 (22.3)
	Bachelor’s Degree or higher	377 (77.7)
Occupation	Unemployed/Student	342 (70.5)
	Employed	143 (29.5)
Monthly Income	<5000 SAR	288 (59.4)
	≥5000 SAR	197 (40.6)

**Table 2:** Demographic factors associated with awareness of toxoplasmosis (n = 485). COR: crude odds ratio; AOR: adjusted odds ratio (multivariate model including all variables); CI: confidence interval; Ref: reference category.

Factor	Aware n (%)	Not Aware n (%)	COR (95% CI)	*p*-Value	AOR (95% CI)	*p*-Value
Gender						
Male	89 (44.3)	112 (55.7)	Ref	—	Ref	—
Female	152 (53.5)	132 (46.5)	1.45 (1.01–2.09)	0.04	1.67 (1.13–2.50)	0.01
Nationality						
Saudi	233 (49.4)	239 (50.6)	Ref	—	Ref	—
Non-Saudi	8 (61.5)	5 (38.5)	1.64 (0.54–5.50)	0.39	2.20 (0.68–7.78)	0.19
Age						
≤30 years	157 (46.9)	178 (53.1)	Ref	—	Ref	—
>30 years	84 (56.0)	66 (44.0)	1.44 (0.98–2.13)	0.06	1.82 (0.80–4.29)	0.16
Marital Status						
Unmarried	154 (47.8)	168 (52.2)	Ref	—	Ref	—
Married	87 (53.4)	76 (46.6)	1.25 (0.86–1.82)	0.25	0.78 (0.39–1.52)	0.46
Residence						
City	136 (48.2)	146 (51.8)	Ref	—	Ref	—
Village	105 (51.7)	98 (48.3)	1.15 (0.80–1.65)	0.45	1.19 (0.82–1.73)	0.35
Education						
High school or less	51 (47.2)	57 (52.8)	Ref	—	Ref	—
Bachelor or higher	190 (50.4)	187 (49.6)	1.14 (0.74–1.75)	0.56	1.06 (0.66–1.69)	0.82
Occupation						
Unemployed	164 (48.0)	178 (52.0)	Ref	—	Ref	—
Employed	77 (53.8)	66 (46.2)	1.27 (0.86–1.88)	0.24	1.10 (0.55–2.17)	0.78
Monthly Income						
<5000 SAR	142 (49.3)	146 (50.7)	Ref	—	Ref	—
≥5000 SAR	99 (50.3)	98 (49.7)	1.04 (0.72–1.49)	0.84	0.98 (0.67–1.43)	0.90

**Table 3:** Knowledge scores across demographic groups (n = 485). SD: standard deviation; CI: confidence interval; MD: mean difference between groups.

Factor	Category	n	Mean ± SD	t-Statistic	*p*-Value	95% CI (MD)
Gender	Male	201	1.6 ± 1.5	4.50	<0.001	0.4–1.0
	Female	284	2.2 ± 1.5			
Nationality	Saudi	472	1.9 ± 1.5	0.28	0.78	0.9–1.1
	Non-Saudi	13	2.0 ± 1.5			
Age	≤30 years	335	1.8 ± 1.5	2.08	0.04	0.0–0.8
	>30 years	150	2.1 ± 1.6			
Marital Status	Unmarried	322	1.8 ± 1.5	1.91	0.06	0.0–1.0
	Married	163	2.1 ± 1.6			
Residence	City	282	1.8 ± 1.5	1.10	0.27	0.2–0.5
	Village	203	2.0 ± 1.5			
Education	High school or less	108	1.9 ± 1.5	0.56	0.57	0.3–0.5
	Bachelor or higher	377	1.9 ± 1.5			
Occupation	Unemployed	342	1.9 ± 1.5	1.18	0.24	0.2–0.6
	Employed	143	2.0 ± 1.6			
Monthly Income	<5000 SAR	288	2.0 ± 1.5	0.61	0.54	0.2–0.4
	≥5000 SAR	197	1.9 ± 1.5			

**Table 4:** Frequency distribution of self-reported risk behaviors (n = 485). SD: standard deviation.

Behavior	Never n (%)	Rarely n (%)	Sometimes n (%)	Often n (%)	Always n (%)	Mean ± SD
Undercooked meat	428 (88.2)	24 (5.0)	22 (4.5)	2 (0.4)	9 (1.9)	1.2 ± 0.7
Raw/undercooked fish	437 (90.1)	29 (6.0)	14 (2.9)	1 (0.2)	4 (0.8)	1.2 ± 0.5
Unwashed produce	351 (72.4)	66 (13.6)	47 (9.7)	15 (3.1)	6 (1.2)	1.5 ± 0.9
Unpasteurized dairy	348 (71.8)	69 (14.2)	43 (8.9)	12 (2.5)	13 (2.7)	1.5 ± 0.9
Hand washing (meat/soil)	78 (16.1)	30 (6.2)	39 (8.0)	34 (7.0)	304 (62.7)	3.9 ± 1.6
Wash produce before eating	66 (13.6)	36 (7.4)	61 (12.6)	64 (13.2)	258 (53.2)	3.8 ± 1.5

## Data Availability

The original contributions presented in this study are included in the article/Supplementary Material. Further inquiries can be directed to the corresponding author.

## Supplementary Materials

The following supporting information can be downloaded at: https://www.mdpi.com/article/10.3390/tropicalmed10110323/s1, Table S1: Strengthening the reporting of observational studies in epidemiology; File S1: The dataset for this study.
